# Hand Hygiene Practices and the Risk of Human Coronavirus Infections in a UK Community Cohort

**DOI:** 10.12688/wellcomeopenres.15796.2

**Published:** 2021-06-22

**Authors:** Sarah Beale, Anne M. Johnson, Maria Zambon, Flu Watch Group, Andrew C. Hayward, Ellen B. Fragaszy

**Affiliations:** 1 UCL Public Health Data Science Research Group, Institute of Health Informatics, UCL, London, NW1 2DA, UK; 2 UCL Institute of Epidemiology and Health Care, UCL, London, WC1E 7HB, UK; 3 UCL Institute of Global Health, UCL, London, WC1E 7HB, UK; 4 Public Health England, London, EC4Y 8AE, UK; 5 NIHR Health Protection Research Unit in Respiratory Infections, Imperial College London, London, W2 1PG, UK; 6 Department of Infectious Disease Epidemiology, London School of Hygiene & Tropical Medicine, London, UK

**Keywords:** coronavirus, hand washing, respiratory hygiene, pandemic, COVID-19, respiratory infection

## Abstract

**Background: **Hand hygiene may mitigate the spread of COVID-19 in community settings; however, empirical evidence is limited. Given reports of similar transmission mechanisms for COVID-19 and seasonal coronaviruses, we investigated whether hand hygiene impacted the risk of acquiring seasonal coronavirus infections.

**Methods: **Data were drawn from three successive winter cohorts (2006-2009) of the England-wide Flu Watch study.  Participants ( *n*=1633) provided baseline estimates of hand hygiene behaviour. Coronavirus infections were identified from nasal swabs using RT-PCR. Poisson mixed models estimated the effect of hand hygiene on personal risk of coronavirus illness, both unadjusted and adjusted for confounding by age and healthcare worker status.

**Results:** Moderate-frequency handwashing (6-10 times per day) predicted a lower personal risk of coronavirus infection (adjusted incidence rate ratio (aIRR) =0.64, *p*=0.04). There was no evidence for a dose-response effect of handwashing, with results for higher levels of hand hygiene (>10 times per day) not significant (aIRR =0.83, *p*=0.42).

**Conclusions: **This is the first empirical evidence that regular handwashing can reduce personal risk of acquiring seasonal coronavirus infection. These findings support clear public health messaging around the protective effects of hand washing in the context of the current COVID-19 pandemic.

## Introduction

The expanding global outbreak of Coronavirus Disease 2019 (COVID-19) demands an evidence-based public health response. Seasonal human coronavirus strains and COVID-19 appear to be transmitted via droplets, direct and indirect contact with infected secretions and, to an unknown extent by aerosol ^1– 4^. Hand hygiene measures are recommended by health authorities and public health experts worldwide to interrupt these transmission mechanisms by preventing viral transfer via contact with infected people and surfaces ^5– 9^. While hand hygiene recommendations are acceptable in a variety of community settings worldwide ^10, 11^ and are widely recommended by health authorities ^5– 9^, evaluation of their effects on the risk of illness in the general population is limited.

Clinical trials of hand hygiene interventions for acute respiratory viral infections in community settings have focused on influenza and yielded mixed results. The balance of evidence suggests small but significant reductions in rates of influenza and influenza-like-illness, likely impacted by the specific community context ^12– 14^. Trial outcomes reflect both the effectiveness of the intervention at altering behaviour as well as the relationship between the behaviour and risk of subsequent illness. As such, observational studies assessing the effect of handwashing on risk of illness are warranted. Limited observational evidence of varying quality has linked increased handwashing with lower risk of influenza and influenza-like illness ^15– 19^, while others have found no relationship ^20– 22^. Larger, population-representative studies are warranted for non-influenza respiratory viruses, particularly coronaviruses, in the current context of the ongoing COVID-19 pandemic.

We aimed to address this gap in the literature using data from a population-based English community cohort study examining transmission of influenza and other respiratory viruses, including seasonal coronaviruses ^23, 24^. As COVID-19 appears to demonstrate similar transmission mechanisms to circulating seasonal coronavirus strains ^1– 4^, understanding the effects of widely-recommended hand hygiene practices may beneficially inform public health campaigns. Understanding the impact of hand hygiene behaviour in community settings is particularly relevant in the absence of an effective vaccine. Consequently, our objective was to investigate whether participants’ frequency of handwashing predicted their overall risk of laboratory-confirmed coronavirus infection.

## Methods

### Study design and procedure

All data were collected as part of Flu Watch, a national household-level prospective cohort study investigating transmission, burden and risk factors associated with influenza and other acute respiratory infections across England. The study methodology has been described in detail elsewhere ^23, 24^. Cohorts were followed up across three winter seasons (2006–2007, 2007–2008, 2008–2009) and the three waves of the 2009 H1N1 pandemic (spring/summer 2009 and winter 2009–2010 and 2010–2011). Data for the present study were drawn from the first three cohort seasons (2006–2009) when coronavirus was regularly monitored in the study.

Demographic data and self-reported hand hygiene were collected at baseline of each season. Participants were followed up weekly by telephone or online to report any acute respiratory illness, and were asked to take a posterior nasal swab in each nostril on the second day of any illness. These samples were requested for all respiratory illnesses during follow up, although in the first winter (2006/7) swabbing was restricted to periods when influenza was known to be circulating. Swabs were placed in a vial of viral transport medium and posted to the Health Protection Agency (HPA) in the same manner as national surveillance samples ^25, 26^. Samples were screened by real-time PCR for a panel of viruses, including seasonal coronaviruses ^27– 30^.

### Participants

Participants were 1633 individuals who participated in the Flu Watch study in any of the three seasons from 2006–2009 and completed a baseline hygiene survey. Participants were randomly selected from patient lists at general practices, and letters were sent inviting their entire household to participate in the study. Households were recruited annually ahead of the influenza season and those who had previously participated were re-invited to the next season in winter 2008–2009. Eligibility criteria were that the full household agreed to follow-up across the whole season and that adults (≥16 years) agreed to have blood samples drawn for other Flu Watch research ^23^. Exclusion criteria were living in a >6-person household, terminal or severe illness or incapacity, and substantial involvement in other ongoing research.

### Measures

***Exposure definitions.*** To assess overall handwashing frequency, the exposure of interest, participants were asked at baseline of each season to “Estimate how many times you washed your hands yesterday”. Frequency of daily handwashing was subsequently categorised as low (≤5 times daily), moderate (6–10 times daily), or high (>10 times daily) guided by literature around influenza-like illness in Western community settings ^20^.

The outcome of interest was whether participants contracted any PCR-confirmed coronavirus infection in a season. Detected coronavirus strains (NL63, OC43, and 229E) were combined into a binary outcome (yes/no coronavirus) as the effect of hand hygiene is believed to be consistent across these strains.

***Covariates.*** Age and healthcare worker status were considered important a-priori potential confounders to be adjusted in analyses due to their relationship both with hygiene practices and with risk of contracting coronavirus infections. Children tend to demonstrate poorer hygiene practices and are more likely to be exposed and to contract acute viral respiratory infections ^12^. Conversely, healthcare workers may be more likely to engage in high-frequency handwashing but also experience more frequent and intense exposure to respiratory viruses, including coronaviruses. Binary indicators (age: child <16 years vs adult ≥ 16 years; healthcare worker status: yes vs no) were created collapsing age and profession categories from the baseline survey.

### Statistical methods

Robust Poisson mixed models were used to estimate the association between hand hygiene and risk of coronavirus infection. Participants’ daily frequency of handwashing was tested as an exposure variable for personal risk of developing PCR-confirmed coronavirus in a season. Hand washing frequency was imputed from the most recent past season for individuals who participated across multiple seasons but did not re-complete the hand hygiene baseline rating.

All models included random effects to account for clustering at the household and individual level (across seasons). Models accounted for person-follow-up-time and were weighted using the inverse of household size to account for the sampling design. Follow-up time was expressed in person-seasons – the proportion of weeks that each participant was active in the study in a given season. Seasons corresponded to the period when coronavirus was estimated to be circulating – defined as the period (in weeks) between the first and last PCR-confirmed case. We excluded participants who participated for a single week. Subsequently, the model was adjusted for confounding by age and healthcare worker status. Results were expressed as incidence rate ratios.

### Ethics statement

The protocol was approved by the Oxford Multi-Centre Research Ethics Committee (06/Q1604/103).

## Results

### Descriptive statistics

***Participants.*** Table 1 reports demographic characteristics and hand hygiene behaviour for the cohort participants included in this study. The majority of included participants (79.85%) were adults over 16 years of age. Each category of handwashing frequency was followed by a considerable subset of the sample (25.88%–39.47%). Participants’ median follow-up time across all seasons was 1.00 person-seasons (IQR 0.94 – 1.00). Further analyses related to clinical details of coronavirus cases and household transmission events are currently in preparation by the Flu Watch group.

**Table 1.** Participant demographic characteristics and hand hygiene behaviour.

Variable		*n* (%)
Age (years)	0–4	95 (5.82%)
	5–15	234 (14.33%)
	16–44	461 (28.23%)
	45–64	574 (35.15%)
	65+	269 (16.47%)
Sex	Female	854 (52.30%)
	Male	779 (47.70%)
Ethnicity	White	1573 (96.33%)
	Non-white	46 (2.82%)
	Unknown	14 (0.86%)
IMD quintile	1 – most deprived	83 (5.08%)
	2	233 (14.27%)
	3	473 (28.97%)
	4	485 (29.70%)
	5 - least deprived	359 (21.98%)
Urban/rural status	Urban	1042 (63.81%)
	Town and fringe	178 (10.90%)
	Village, hamlet and isolated dwellings	409 (25.05%)
	Unknown	4 (0.24%)
Area of England	North	213 (13.04%)
	West Midlands	152 (9.31%)
	East and East Midlands	289 (17.70%)
	London	103 (6.31%)
	South East	259 (15.86%)
	South West	617 (37.78%)
Household Size ( *n* people)	1	91 (5.57%)
	2	632 (38.70%)
	3	302 (18.49%)
	4	392 (24.00%)
	5+	216 (13.23%)
Healthcare Worker	Yes	89 (5.45%)
	No	1544 (94.55%)
How many times did you wash your hands yesterday? *	0–5	499 (25.88%)
	6–10	761 (39.47%)
	>10	668 (34.65%)

*Note: handwashing frequencies include all seasons of participant follow-up

Table 2 reports hand hygiene frequency by age group and healthcare worker status, the a-priori potential confounders included in the statistical analyses. The majority of children between 0-4 (71.58%) and 5-15 (52.99%) reported low-frequency handwashing, which was reported by a lower proportion of participants across adult age groups (14.11-21.69%). Low frequency handwashing was reported by 26.62% of non-healthcare workers versus 8.99% of healthcare workers.

**Table 2.** Hand Hygiene Frequency by Age Group and Healthcare Worker Status.

		Wash hands – previous day: n (row %)		
		0–5 times	6–10 times	11+ times
Age (years)	0–4	68 (71.58)	25 (26.32)	2 (2.11)
	5–15	124 (52.99)	96 (41.03)	14 (5.98)
	16–44	100 (21.69)	194 (42.08)	167 (36.23)
	45–64	81 (14.11)	243 (42.33)	250 (43.55)
	65+	46 (17.10)	103 (38.29)	120 (44.61)
Healthcare Worker	Yes	8 (8.99)	26 (29.21)	55 (61.80)
	No	411 (26.62)	635 (41.13)	498 (32.25)

### Hygiene behaviours and risk of coronavirus infection

Table 3 reports the results of models estimating the relationship between frequency of handwashing and participants’ risk of coronavirus infection. Moderate-frequency handwashing was associated with significantly reduced risk of contracting coronavirus compared to low handwashing, (adjusted IRR= 0.64, 95% CI: 0.42, 0.99, *p*=0.046). For higher intensity handwashing there was no significant effect (adjusted IRR = 0.83, 95% CI (0.53, 1.30) *p*=0.42.

**Table 3.** Handwashing frequency and participant risk of coronavirus infection.

				Risk - Overall			
				IRR (95% CI)			
		*n -* No Infection	*n* -Confirmed Infection	Baseline	*p*	Adjusted	*p*
Wash hands – previous day	0–5 times	443	56	..	..	..	..
	6–10 times	706	55	0.65 (0.43, 0.99)	0.04	0.64 (0.42, 0.99)	0.046
	11+ times	606	62	0.83 (0.55, 1.25)	0.37	0.83 (0.53, 1.30)	0.42

## Discussion

This study aimed to assess the relationship between hand hygiene and the risk of contracting seasonal coronavirus infection in an English community cohort. Moderate-frequency handwashing was associated with reduced overall risk of coronavirus infection. These results provide support for public health messages endorsing regular handwashing for personal protection. Given that COVID-19 appears to demonstrate similar transmission mechanisms to seasonal coronaviruses ^1– 4^, these findings indicate that public health campaigns to increase uptake of regular hand hygiene in community settings are warranted during the current pandemic.

We show a valuable reduction in the risk of acquiring coronavirus infection in moderate-frequency handwashers. This is consistent with the limited observational literature on influenza and influenza-like-illness in community settings ^15– 19^. The study did not demonstrate a clear dose-response relationship for the protective effect of hand hygiene frequency, with the strongest personal protective effect seen in the moderate-level hand hygiene group and a non-significant protective effect in the highest-frequency hand hygiene group. However, numbers in the highest-frequency handwashing group were lower, limiting power. It is also possible that there is residual confounding. For example, while we adjusted for healthcare worker status, those working in other public-facing professions may be more likely to be high-frequency handwashers and may have increased exposure to coronavirus, which would have mitigated against seeing a protective effect of high-frequency handwashing. The context of handwashing and compliance with recommended handwashing procedures are likely also important. Both longer duration of handwashing and the context of handwashing (e.g. after shaking hands or before eating) have been associated with lower overall risk of influenza or influenza-like-illness in previous studies ^15, 18^. Only handwashing frequency, and not duration and context of handwashing, were assessed in the behavioural questionnaire used in the current study. Further research into the relationship between hand hygiene practices and risk of coronavirus infections should include all these elements of hand hygiene behaviour.

Despite Flu Watch being the one of the largest cohort studies of respiratory infection, the relatively small number of coronavirus diagnoses limited power and ability to control for confounding. Examining asymptomatic cases was not feasible as samples were only taken from symptomatic cases. Data were also limited to the winter, though included coronavirus strains demonstrate winter seasonality in temperate climates ^31, 32^. Hand hygiene was self-reported and therefore may have been affected by social desirability and recall biases. The handwashing frequency item was only measured at baseline and was estimated from the previous 24-hour period so may not reflect usual behaviour for all participants, or behaviour at time of exposure.

This study was the first to examine the personal protective effect of hand hygiene on respiratory coronavirus infections in a community setting. The findings are likely to be generalisable to similar high-income countries. The generalisability of the findings to other coronavirus infections, notably COVID-19, also depends on similarity of epidemiological features. Given the indication of similar transmission mechanisms for COVID-19, corresponding protective effects of handwashing are highly plausible. Strength of effects, however, may depend on the transmissibility of the pathogen and levels of population immunity. Nevertheless, our findings represent important empirical evidence of the value of hand washing, supporting public communication about the importance of this behaviour. Hand washing is minimally disruptive and socially acceptable in a variety of community settings and has an important role in raising awareness and slowing transmission alongside measures such as physical and social distancing ^10, 11^.

## Data availability

### Source data

Open Science Framework: Hand Hygiene Practices and the Risk of Human Coronavirus Infections in a UK Community Cohort. https://doi.org/10.17605/OSF.IO/UGRMY ^33^.

This project contains the following underlying data:

- FluWatch_HandHygiene_C0V_HygRisk.dta (hand hygiene data; DTA format).
- HygRisk.csv dta (hand hygiene data; CSV format).

Data are available under the terms of the Creative Commons Attribution 4.0 International license (CC-BY 4.0).
