# Supplementary material for: Hand Hygiene Practices and the Risk of Human Coronavirus Infections in a UK Community Cohort
Source: Wellcome Open Res. 2021 Jun 22;5:98. Originally published 2020 May 20. [Version 2] doi: 10.12688/wellcomeopenres.15796.2 (PMC8243225; doi:10.12688/wellcomeopenres.15796.2)
Supplement: Peer review history

# Peer review: Reviewer response for version 2

**DOI:** 10.21956/wellcomeopenres.18769.r44599

**Published:** 2021-06-29

**Authors:** Peggy Or

**Affiliations:** 1 The Education University of Hong Kong, Hong Kong, Hong Kong

I have no further comment. Approved for indexing.

Is the work clearly and accurately presented and does it cite the current literature?

Yes

If applicable, is the statistical analysis and its interpretation appropriate?

Partly

Are all the source data underlying the results available to ensure full reproducibility?

Yes

Is the study design appropriate and is the work technically sound?

Yes

Are the conclusions drawn adequately supported by the results?

Partly

Are sufficient details of methods and analysis provided to allow replication by others?

Yes

Reviewer Expertise:

Infection control.

I confirm that I have read this submission and believe that I have an appropriate level of expertise to confirm that it is of an acceptable scientific standard.

# Peer review: Reviewer response for version 1

**DOI:** 10.21956/wellcomeopenres.17322.r38956

**Published:** 2020-07-06

**Authors:** Peggy Or

**Affiliations:** 1 The Education University of Hong Kong, Hong Kong, Hong Kong

- If applicable, is the statistical analysis and its interpretation appropriate: **Partly.**

There was no statistical analysis on demographic data and in Table 1. no interpretation on demographic characteristics and hand hygiene behaviour. There was no further elaboration on hand washing frequency and participants’ demographic data and confirmed cases.

 The result section was thin and it was difficult to draw solid conclusion. The arguments in the discussion were not drawn from the study findings such as “both longer duration of handwashing and the context of handwashing have been associated with lower overall risk of influenza or influenza-like-illness”. However, there were no such information on duration and context of handwashing in the study.

- Are the conclusions drawn adequately supported by the results? **Partly.**

The conclusion drawn from handwashing can reduce personal risk of acquiring seasonal coronavirus infection, it was general. It did not further explain why only moderate-frequency handwashing was associated with significantly reduced risk of contracting coronavirus compared to low or high frequency handwashing.

Is the work clearly and accurately presented and does it cite the current literature?

Yes

If applicable, is the statistical analysis and its interpretation appropriate?

Partly

Are all the source data underlying the results available to ensure full reproducibility?

Yes

Is the study design appropriate and is the work technically sound?

Yes

Are the conclusions drawn adequately supported by the results?

Partly

Are sufficient details of methods and analysis provided to allow replication by others?

Yes

Reviewer Expertise:

Infection control.

I confirm that I have read this submission and believe that I have an appropriate level of expertise to confirm that it is of an acceptable scientific standard, however I have significant reservations, as outlined above.

# Response

**Published:** 2021-06-17

**Authors:** Sarah Beale

**Affiliations:** University College London, UK

*Many thanks for your helpful comments on the manuscript. We have carefully considered your feedback and present a point-by-point response to each comment below.*

 "There was no statistical analysis on demographic data and in Table 1. no interpretation on demographic characteristics and hand hygiene behaviour. There was no further elaboration on hand washing frequency and participants’ demographic data and confirmed cases."

*Thank you for raising this point. We have now added a new table (Table 2) which presents hand hygiene frequency by age group and healthcare worker status, the demographic covariates included in statistical analyses; corresponding text is in Paragraph 2 of the Results. Table 1 was intended to describe the demographic composition of the sample, and the full table was not stratified by hand hygiene frequency as not all demographic variables were included in the main analyses due to limited power (as highlighted in Paragraph 2 of the discussion) and not being selected as most relevant potential confounders a-priori. The main analyses (Table 3) were adjusted for a-priori selected demographic variables (age and healthcare worker status) and regression coefficients of the demographic covariates were not reported separately to avoid the Table 2 fallacy (https://doi.org/10.1093/aje/kws412). Investigation into demographic features of confirmed cases is planned for separate analyses (please see Descriptive Statistics paragraph 1).*

 "The result section was thin and it was difficult to draw solid conclusion. The arguments in the discussion were not drawn from the study findings such as “both longer duration of handwashing and the context of handwashing have been associated with lower overall risk of influenza or influenza-like-illness”. However, there were no such information on duration and context of handwashing in the study."

*Thank you for highlighting this issue. We included discussion about duration and context to indicate that these were other important hand hygiene-related factors that have been associated with risk of influenza or influenza-like-illness in the literature but could not be assess in the present study as they were not measured. We have now clarified this point in Paragraph 2 of the discussion, as follows: “Both longer duration of handwashing and the context of handwashing e.g. after shaking hands or before eating – have been associated with lower overall risk of influenza or influenza-like-illness in previous studies (15,18). Only handwashing frequency, and not duration and context of handwashing, were assessed in the behavioural questionnaire used in the current study. Further research into the relationship between hand hygiene practices and risk of coronavirus infections should include all these elements of hand hygiene behaviour.”*

 "The conclusion drawn from handwashing can reduce personal risk of acquiring seasonal coronavirus infection, it was general. It did not further explain why only moderate-frequency handwashing was associated with significantly reduced risk of contracting coronavirus compared to low or high frequency handwashing."

*We discuss this finding and potential explanations, including low power and residual confounding, in Paragraph 2 of the discussion. To clarify that this is the topic of discussion in this paragraph, we have rephrased the following sentence leading into this discussion (Discussion paragraph 2, sentence 3): “The study did not demonstrate a clear dose-response relationship for the protective effect of hand hygiene frequency, with the strongest personal protective effect was seen in the moderate-level hand hygiene group and a non-significant protective effect in the highest-frequency hand hygiene group.”*

# Peer review: Reviewer response for version 1

**DOI:** 10.21956/wellcomeopenres.17322.r38788

**Published:** 2020-05-27

**Authors:** Colum P. Dunne

**Affiliations:** 1 University of Limerick, Limerick, Ireland

This report is timely and relevant in developing insight into hand hygiene practices, and benefits, in community settings with relevance to the SARS-CoV2 (COVID-19) pandemic. Not prospective, the study utilises existing data generated with respect to seasonal influenza in 2006-2009. Specifically, the authors focus on self-declared hand hygiene (general hand-washing) by community-based participants, and subsequent incidence of flu, with supporting confirmation of infection via nasal swabs.

 Overall, the conclusions from this study are measured, and the biases involved are identified. In particular, the challenges associated with self-declaration of practices and memory-based estimation. The authors also discuss the issue of lack of appropriate hand hygiene training or education. This is especially topical as, in the current pandemic, there has been considerable promotion of good practices and an adoption, to some degree, of effective hand-washing procedures and the WHO 5 moments...adapted for community settings and routine living outside of hospitals. The shortage of alcohol gel sanitisers and wipes speaks to this change.

 One statement by the Authors is slightly challenging; the conclusion that this study is generalisable "probably also in low- and middle-income countries". This is an assumption that does not consider clean water or soap/surfactant/alcohol gel availability in countries where supply chains, economics, nutrition and basic living conditions do not mirror developed countries. 

 Otherwise, this is a timely and useful paper.

Is the work clearly and accurately presented and does it cite the current literature?

Yes

If applicable, is the statistical analysis and its interpretation appropriate?

Not applicable

Are all the source data underlying the results available to ensure full reproducibility?

No source data required

Is the study design appropriate and is the work technically sound?

Yes

Are the conclusions drawn adequately supported by the results?

Yes

Are sufficient details of methods and analysis provided to allow replication by others?

Yes

Reviewer Expertise:

Translational research, microbiology, infectious disease, outbreaks, management, characterisation.

I confirm that I have read this submission and believe that I have an appropriate level of expertise to confirm that it is of an acceptable scientific standard.

# Response

**Published:** 2021-06-17

**Authors:** Sarah Beale

**Affiliations:** University College London, UK

*Many thanks for your positive feedback about the manuscript. Following on from your comments, we have now revised the statement about generalisability in the discussion to read: “The findings are likely to be generalisable to similar high-income countries.”*
